# Inotuzumab ozogamicin in B-cell precursor acute lymphoblastic leukemia: efficacy, toxicity, and practical considerations

**DOI:** 10.3389/fimmu.2023.1237738

**Published:** 2023-08-03

**Authors:** Jeremy D. Rubinstein, Maureen M. O’Brien

**Affiliations:** ^1^ Department of Pediatrics, University of Cincinnati College of Medicine, Cincinnati, OH, United States; ^2^ Division of Oncology, Cancer and Blood Diseases Institute, Cincinnati Children’s Hospital Medical Center, Cincinnati, OH, United States

**Keywords:** inotuzumab ozogamicin, BCP-ALL, acute lymphoblastic leukemia, antibody drug conjugate, CD22, relapse/refractory, sinusoidal obstruction syndrome

## Abstract

Inotuzumab ozogamicin (InO) is an antibody drug conjugate composed of a humanized monoclonal antibody targeting the cell surface receptor CD22 coupled to a cytotoxic calicheamicin payload *via* an acid labile linker. InO has shown significant activity in relapsed and refractory B-cell precursor acute lymphoblastic leukemia (BCP-ALL) in both single agent and combination chemotherapy regimens in adult and pediatric trials. Its use in newly diagnosed elderly patients has also been established while clinical trials investigating its use in newly diagnosed pediatric patients and fit adults are ongoing. Notable toxicities include sinusoidal obstruction syndrome (SOS), particularly in patients who undergo hematopoietic stem cell transplantation (HSCT) after InO as well as myelosuppression and B-cell aplasia which confer increased infection risk, particularly in combination with cytotoxic chemotherapy. In the relapsed/refractory (R/R) setting, the planned subsequent curative therapy modality must be considered when using InO to mitigate SOS risk if proceeding to HSCT and account for potential B-cell aplasia if proceeding to chimeric antigen receptor CAR-T therapy. Studies exploring mechanisms of resistance or failure of InO are ongoing but modulation or loss CD22 expression, alternative CD22 splicing, and high Bcl-2 expression have been implicated. In this review, we will summarize the currently available data on InO, with an emphasis on pediatric trials, and explore future directions including combinatorial therapy.

## 1 Introduction

Outcomes for children and adolescents with B-cell acute lymphoblastic leukemia (BCP-ALL) continue to improve, with a 5-year overall survival (OS) rate for pediatric patients surpassing 90% (1, 2). However, outcomes remain inferior for infants and adolescent and young adult (AYA) patients as well as those with high-risk cytogenetic or molecular features such as hypodiploidy, Philadelphia chromosome positive (Ph+) and Philadelphia chromosome-like (Ph-like) (3–5). Recent efforts to improve outcomes by further intensification of cytotoxic chemotherapy have been unsuccessful in part due to excessive toxicity (6). Patients with Down syndrome have inferior outcomes due to a combination of genomic factors and chemotherapy-related toxicity (7). For children who experience early first relapse or second or greater relapse, outcomes remain poor despite the availability of HSCT and chimeric antigen receptor T- cell (CAR-T) therapy (8). As a result, novel agents and therapy combinations are still needed for high-risk patients in the both the upfront and R/R setting.

CD22 is a sialic acid-binding immunoglobulin-like lectin (Siglec) and member of the immunoglobulin superfamily that is expressed on the cell surface of normal B-lymphocytes throughout all stages of B-cell development except plasma cells; it is not expressed on hematopoietic stem cells or other tissues (9). CD22 is expressed on the cell surface of the majority of BCP-ALL; among 163 pediatric patients with R/R BCP-ALL, CD22 was detected on at least 90% of blasts in 155 cases (95%). The notable exception was blasts from patients with 11q23 (KMT2A) rearrangements in which six of 21 patients had sub-populations of blasts lacking CD22 expression (range 22-82% CD22-positive) (10). In a cohort of 142 adults with newly diagnosed ALL, surface CD22 (sCD22) was present on >90% of blasts in 55% of patients, on 51-90% of blasts in 16% of patients, on 11-50% of blasts in 14% of patients, and on 0-10% of blasts in 4% of patients (11). Like CD19, the broad expression of CD22 on BCP-ALL and restriction to B-cells makes both attractive therapeutic targets. In contrast, CD20 is expressed universally on mature B cells and less commonly on B-precursor cells; 30-50% of BCP-ALL cases have CD20 expression on >20% of blasts (12). Trials in adults have demonstrated some benefit from the addition of rituximab to chemotherapy for patients with CD20+ BCP-ALL, but given the significant proportion of patients without CD20 expression, this strategy has not been pursued in pediatric BCP-ALL (13).

**Figure 1:**
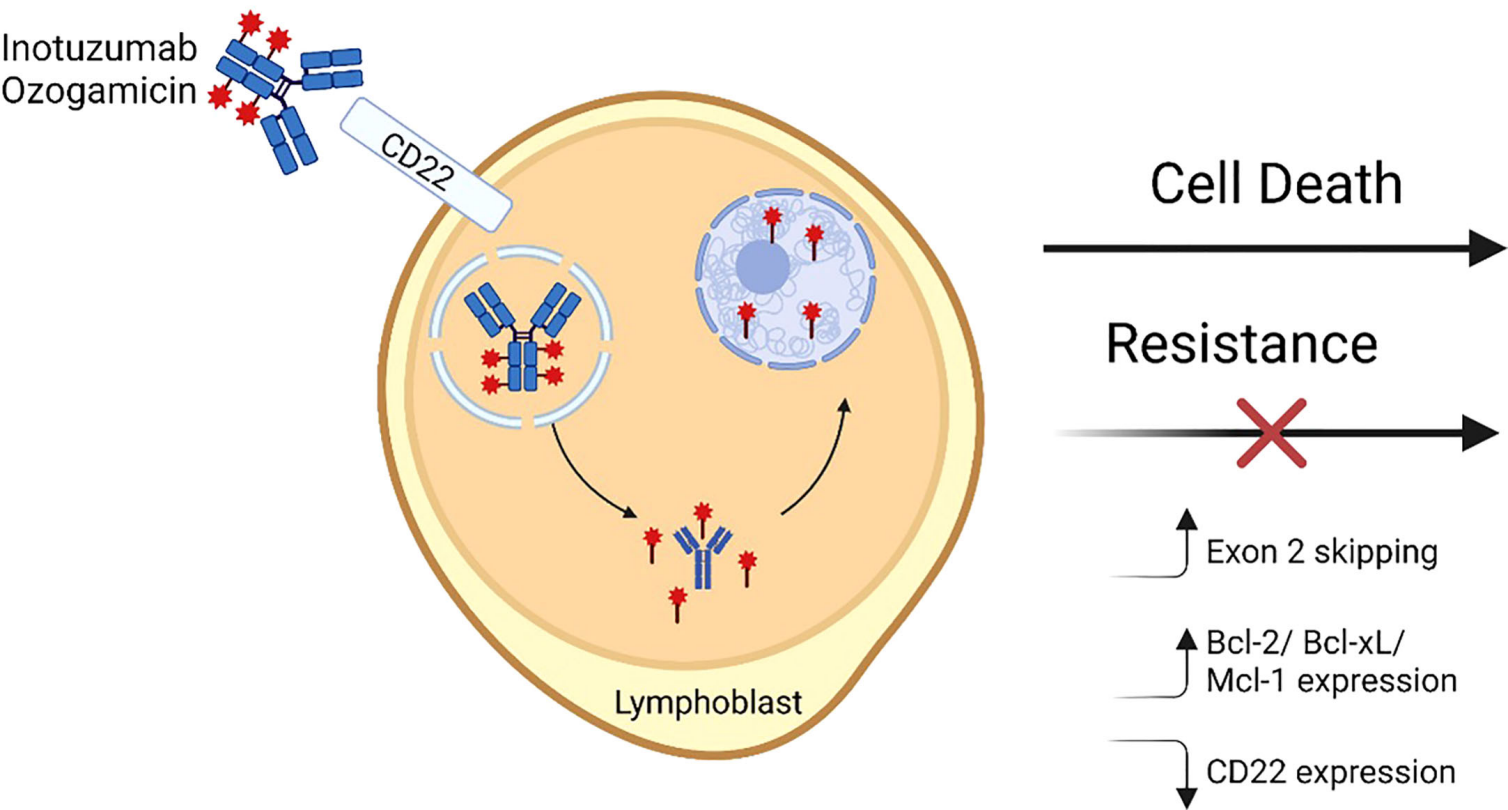
Inotuzumab ozogamicin mechanism of action and resistance. A fully humanized CD22 monoclonal antibody is coupled to a cytotoxic antibiotic calicheamicin (red star) *via* an acid labile linker. The antibody binds cell surface CD22 and then is internalized into lysosomal vesicles where the linker undergoes hydrolysis and releases the calicheamicin molecules which bind DNA in the minor groove in the minor groove. Subsequently induced structural changes lead to the formation of hydrogen abstracting diradical that ultimately causes DNA breaks and apoptosis. Mechanisms that have been shown to lead to resistance to InO include alterations in CD22 RNA splicing leading to increased exon 2 skipping, increased expression of pro-apoptotic molecules, and/or decreases in CD22 expression. Figure made with BioRender.

For CD19 and CD22, targeted therapies include chimeric antigen receptor (CAR) T-cells, antibody drug conjugates (ADC), and bispecific T-cell engaging antibodies. CD19-targeting FDA-approved immunotherapies for BCP-ALL include blinatumomab and tisagenlecleucel; CD22 and dual targeting (CD19/CD22) CAR T-cell therapies are under investigation and available through clinical trials (14–16). ADC therapies depend upon the ability of the antibody to bind the receptor, internalize, and release a cytotoxic payload intracellularly (**Figure 1**) (17). CD22 was found to be an ideal ADC target based on its internalization properties and lack of extracellular shedding (18). Notably, CD22 immunotoxins have significantly lower half maximal inhibitory concentration (IC_50_) than CD19 immunotoxins despite similar binding affinity and at least 10-fold lower CD22 site density on the cell surface, presumably due to rapid receptor-mediated endocytosis at a rate 2-3 fold higher than the number of CD22 molecules on the cell surface (19). This finding may explain in part why ADCs targeting CD22 have been more successful clinically than those targeting CD19.

Inotuzumab ozogamicin (InO), initially known as CMC-544, is an ADC in which a fully humanized monoclonal antibody targeting CD22 is coupled with a cytotoxic calicheamicin antitumor antibiotic by an acid labile linker. Upon binding and internalization, the molecule is internalized into lysosomal vesicles where hydrolysis of the acid labile linker liberates the calicheamicin. The toxin then binds DNA in the minor groove and undergoes structural changes leading to a hydrogen abstracting diradical that ultimately causes DNA breaks and apoptosis (20, 21). Considering strong preclinical data, clinical trials were developed in the first decade of the 2000s for adults with both R/R CD22+ BCP-ALL and B-cell non-Hodgkin lymphoma (B-NHL). BCP-ALL studies will be discussed in depth later in this review. InO is the only ADC FDA-approved for the treatment of BCP-ALL (in adults); in contrast, CD19-targeting ADCs have shown promise in B-NHL but have not demonstrated sufficient efficacy in BCP-ALL (17).

## 2 Pharmacology

The first-in-human phase I study of InO, conducted in adults with B-NHL, found a maximum tolerated dose (MTD) of 1.8 mg/m^2^ given intravenously (IV) every 4 weeks with dose limiting toxicity (DLT) of thrombocytopenia (22). Initial *in vitro* studies using pediatric primary BCP-ALL cells demonstrated that while CD22 expression was necessary for InO uptake, there was no requirement for subsequent renewed CD22 expression to achieve sufficient intracellular levels, suggesting that high doses may not be necessary for therapeutic benefit (23). In part based on this data, subsequent adult BCP-ALL trials fractionated the total InO dose to provide 0.8 mg/m^2^ on day 1 followed by 0.5 mg/m^2^ on days 8 and 15 of an initial cycle (24). In a phase 2 study in adults with R/R BCP-ALL, patients treated with single dose InO at 1.8mg/m^2^ on day 1 had higher peak levels compared to those treated with weekly fractionated dosing, but peak InO levels were not associated with response. In contrast, higher cumulative area under the curve (AUC) levels were associated with increased likelihood of complete response (CR) and AUC levels were similar between fractionated and single dose regimens.

The benefit of dose fractionation in BCP-ALL, as compared to B-NHL where fractionation was not felt to be needed, is supported by a pharmacokinetic/pharmacodynamic (PK/PD) model incorporating both preclinical and clinical patient data (25). InO PK parameters are best described by a two-compartment model with linear and time-dependent clearance such that early clearance in the setting of gross disease burden is predominantly receptor-mediated and drug is cleared rapidly by CD22 binding and internalization with a half-life (t_1/2_) of 6 days for the first dose. However, as disease burden decreases, receptor-mediated clearance becomes negligible, and t_1/2_ lengthens to 13.8 days at steady state (26). This model also suggested that InO PK and N-ac-gamma-calicheamicin DMH release are more sensitive and useful predictors of outcomes in InO recipients than CD22 expression. Data from 243 adult patients treated with InO showed that dose exposure was significantly correlated with the achievement of both CR and MRD negativity (27). A more robust PK model was developed by using a population PK approach pooling samples from multiple adult clinical trials; both body surface area (BSA) and the baseline percentage of blasts in the peripheral blood were covariates for the time-dependent clearance components. However, the magnitude of change for PK parameters due to these covariates was not deemed to be clinically relevant and no dose adjustments were indicated based on these covariates (26). Additionally, other demographic factors (e.g., age, race, and sex) and measures of renal and hepatic function did not impact InO PK. In a study applying this model to samples from 53 pediatric patient with BCP-ALL treated with InO, the same covariates were identified (28). Of note, ADCs in general, including InO, do not cross the blood-brain barrier and there is no available data to support its use for central nervous system ALL (29, 30).

## 3 Toxicities

### 3.1 Sinusoidal obstruction syndrome

Previously known as veno-occlusive syndrome (VOD), sinusoidal obstruction syndrome (SOS) is a clinical syndrome that can be seen after many chemotherapies but occurs most frequently after HSCT. SOS is characterized by a constellation of symptoms including hepatomegaly, hyperbilirubinemia, right upper quadrant pain, ascites as well as thrombocytopenia and coagulopathy and is due to injury of the sinusoidal endothelium (31). Diagnosis is generally supported by clinical symptoms and findings of reversal of portal venous flow by Doppler ultrasound (32). In addition to supportive care measures like transfusions and diuresis, SOS can be treated with high dose intravenous steroids and/or defibrotide; mortality rates can be high especially in cases of severe SOS with multiorgan failure (33, 34). Expert consensus recommendations regarding the diagnosis and management of SOS in children, adolescents, and young adults have been published (35).

Drug-induced liver injury and SOS are well-documented with InO therapy, and postulated to be due to non-specific uptake of InO by liver sinusoidal endothelial cells (SECs) given the lack of CD22 expression within the normal liver (36). Notably, SOS is also an important side effect of gemtuzumab ozogamicin (GO), a CD33-calicheamicin ADC approved for the treatment of acute myeloid leukemia (AML), suggesting that this toxicity is related to the common cytotoxic payload calicheamicin and not specific to either CD22 or CD33 targeting (37, 38). This was demonstrated in a study of a non-binding ADC containing the same linker and calicheamicin payload as InO and GO in cynomolgus monkeys (39). Liver evaluation three days after non-binding ADC exposure demonstrated midzonal degeneration and loss of SECs associated with platelet accumulation in sinusoids. Subsequent evaluation on day 63 showed recovery with sinusoidal capillarization and sinusoidal dilation with hepatocellular atrophy, consistent with early SOS. Notably, there was no effect on bone marrow megakaryocytes or activation of platelets in peripheral blood, suggesting that the mechanism for thrombocytopenia following calicheamicin ADC therapy is related to endothelial injury, activation, and platelet sequestration rather than marrow suppression.

Our understanding of risk factors associated with the development of SOS is limited, with the primary risk factor being HSCT after InO therapy. There have been no clinical trials to date in which details of post-InO HSCT were uniform and dictated as part of the trial protocol; rather, patients treated with InO received subsequent HSCT at the discretion of the treating physician, including the timing, conditioning regimen, donor selection, selection of bridging therapy, use of SOS prophylaxis, and selection of GVHD prophylaxis regimen. Thus, SOS risk factors and outcomes for patients receiving InO after HSCT are limited to descriptive results which are summarized below and in **Table 1**.

**Table 1:** SOS rates and risk factors in completed prospective clinical trials of inotuzumab ozogamicin in relapsed/refractory BCP-ALL.

Trial	Age	Treatment	N	SOS during or after InO without HSCT	N with HSCT after InO	SOS during HSCT	SOS Risk factors	Ref
Pfizer Phase 1/2	Adult	InO 1.2-1.8 mg/m^2^ fractionated dosing	72	2	24	2	None	47
MDACC Phase 2	Adult	InO Cohort 1: 1.3-1.8mg/m^2^ every 3-4 weeks Cohort 2: 1.8mg/m^2^ fractionated dosing	90	0	36	6	Unfractionated dosing; dual alkylator conditioning	24
INO-VATE	Adult	InO 1.8mg/m^2^ fractionated dosing	164	5	77	17	Dual alkylator conditioning; elevated pre-HSCT bilirubin	48
S1312	Adult	InO fractionated dosing + CVP	48	0	13	3	None	49
MDACC Combination Phase 2	Adult	MiniCVD + InO 1-1.8mg/m^2^ on day 3 + versus InO 0.6-0.9mg/m^2^ fractionated on day 2 and 8	96	3	44	7	Unfractionated dosing	50
COG AALL1621	Child	InO 1.8mg/m^2^ fractionated dosing	46	0	21	6	None	44
ITCC-059	Child	InO 1.4-1.8mg/m^2^ fractionated dosing	52	3	23	6	Shorter time between InO and HSCT	45, 51

COG, Children’s Oncology Group; CVP, cyclophosphamide, vincristine, and prednisone; HSCT, hematopoietic stem cell transplantation; ITCC, Innovative Therapies for Children with Cancer; InO, inotuzumab ozogamicin; MDACC, MD Anderson Cancer Center; SOS, sinusoidal obstruction syndrome.

An increased risk for SOS with InO was first reported in an analysis of 26 adult patients treated with InO at MD Anderson Cancer Center (MDACC) who subsequently underwent allogeneic HSCT. Five patients developed SOS, which was fatal in all cases. Unfractionated InO had last been administered at a median of 40 days prior to the start of the HSCT conditioning regimen (47). On the phase 3 adult INO-VATE trial, which utilized fractionated InO dosing of 1.8 mg/m^2^ in the first cycle followed by 1.5 mg/m^2^ per cycle once a CR was achieved, SOS were seen in 15 of 139 (10.8%; any grade) and 13 of 139 (9.4%, grade 3 and higher) patients on the InO arm compared to just one of 120 (0.8%) patients on the standard of care chemotherapy arm (48). Twenty-two percent of patients on the InO arm who proceeded to HSCT developed SOS compared to 1% of patients on the standard of care chemotherapy arm, with five patients having fatal SOS. Meanwhile, amongst patients who did not proceed to HSCT, 3% of InO recipients developed SOS compared to none in the standard of care chemotherapy arm. Pooled data from INO-VATE with the phase 1/2 multicenter 1010 study showed that 18.8% of patients who received InO and then proceeded to HSCT developed SOS which was fatal in 5 of 19 (26%) cases. As compared to the 82 InO recipients who proceeded to HSCT who did not develop SOS, there was no significant difference in median time from last InO dose to HSCT (49). Recent analysis from MDACC of 245 patients found that only subsequent HSCT was associated with higher risk on a univariate analysis, although this became non-significant on a multivariate analysis (50).

Pediatric data is similar; in a multicenter retrospective study of 51 patients who received InO on a compassionate access basis, no patients developed SOS during InO therapy but 11 of 21 recipients who proceeded to HSCT developed SOS (44). On the prospective Children’s Oncology Group (COG) AALL1621 trial of single-agent InO, six of 21 patients who proceeded to HSCT developed grade 3 or higher SOS. No statistically significant associations with SOS were seen on this study although analysis was limited small sample size (45). On the ITCC-059 European prospective pediatric phase 1 study, no SOS occurred in the seven patients who proceeded to HSCT, although SOS did occur in two patients who underwent subsequent multiagent chemotherapy for relapsed/refractory disease (51). On the phase 2 portion of this trial, seven of 28 patients developed SOS, six during post-InO and one in a patient who had prior HSCT before InO therapy (41). Dosing schedules were similar in all three of these pediatric reports.

In terms of other risk factors, InO exposure is positively correlated with the risk for SOS in adults (27). In multivariate analysis on INO-VATE, the use of dual alkylator conditioning regimens for HSCT and elevated pre-HSCT bilirubin levels were significant covariates for development of SOS (52). In a study of 47 patients treated at City of Hope who proceeded to HSCT after InO exposure, there was no significant difference between those with and without SOS regarding median duration between InO and HSCT, utilization of myeloablative conditioning, use of total body irradiation, or disease status at HSCT. Only the use of sirolimus as graft-vs-host disease prophylaxis was associated with SOS risk on univariate analysis (53).

On the COG AALL1732 study (NCT 03959085) which is investigating the incorporation of two blocks of InO into the modified Berlin-Frankfurt-Munster (mBFM) chemotherapy backbone for newly diagnosed high-risk pediatric and AYA BCP-ALL, in two consecutive safety phases seven of 48 (14.5%) of patients randomized to the InO arm developed SOS compared to one of 50 patients (2%) of patients on the control arm (54, 55). For the patients on the InO arm, six of seven cases occurred during the Delayed Intensification (DI) phase of therapy, and ranged from grade 2 (mild symptoms requiring no intervention in three cases) to grade 3 or 4 (treated with defibrotide in three cases); five of the six cases occurred proximate to thioguanine administration in DI which is notable given the known association of thiopurines with SOS (56). Future study amendment will omit thioguanine on the InO arm to mitigate this risk.

Prophylactic strategies to decrease the risk of SOS, particularly in the setting of HSCT, remain under investigation as no clearly effective measures are currently available. One expert panel recommended avoiding dual alkylator conditioning regimens, avoiding azole or other know hepatotoxic medications in combination with high dose alkylators, and prophylactic administration of ursodiol to patients recently exposed to InO (57). In one analysis of patients who received ursodiol prophylaxis for HSCT after InO or GO therapy, there was no different in hepatotoxicity compared to those who did not receive ursodiol but the sample size for both groups was small (58). The benefit of prophylactic defibrotide is unclear as reports on its use in this setting are limited to small case series (59). In a randomized multicenter phase 3 trial for adult and pediatric patients undergoing HSCT and considered at high risk for SOS, no benefit was observed for defibrotide prophylaxis over best standard of care; approximately 25% of patients on each arm had received prior calicheamicin-based ADC and in *post hoc* analysis SOS-free survival rates at day 30 and day 100 were similar between patients with and without previous calicheamicin-based ADC exposure (40).

### 3.2 Hematologic toxicity

Hematologic toxicity has been seen on all trials using InO. In terms of single agent studies, on the pediatric AALL1621 trial for patients with multiply R/R BCP-ALL using the FDA-approved fractionated dosing for adult ALL, hematologic DLT (failure to achieve absolute neutrophil count (ANC) ≥ 500 cells/µL and transfusion-independent platelet count of ≥ 20,000 cells/µL not due to malignant infiltration for greater than or equal to 42 days from the start of a cycle) occurred in 7/28 (25%) of patients with complete response (CR) or CR with incomplete count recovery (CRi) (45). On the phase 1/2 adult 1010 study for R/R BCP-ALL, 10% of patients required dose reductions due to treatment emergent adverse events, most commonly from thrombocytopenia and neutropenia while 28% of patients required dose delays for these reasons (60). Of note, on INO-VATE, the percentage of patients with grade 3/4 thrombocytopenia was lower in the InO group than those who received standard of care chemotherapy (high-dose cytarabine-based regimens) (48).

Regarding clinical trials using InO in combination with chemotherapy, in a study of elderly patients receiving InO with mini-hyper CVD (dose-reduced cyclophosphamide, vincristine, dexamethasone alternating with methotrexate, cytarabine) for upfront leukemia, 79% of patients had prolonged thrombocytopenia beyond six weeks at some point during their therapy (46). In the ITCC-059 pediatric phase 1 trial in R/R patients, when InO was combined with dexamethasone and vincristine, grade 3/4 thrombocytopenia was reported in 19 of 37 (63.3%) of patients (41).

### 3.3 Infections

In single agent studies, infectious toxicity from InO has been relatively low. On INO-VATE, the grade 3 febrile neutropenia rate was 11% in InO recipients as compared to 18% in the standard of care arm while sepsis rates were 2% and 5% in those same groups. On the pediatric AALL1621 study, 29% of patients had febrile neutropenia in cycle 1 but there were zero cases in cycle 2 and a low sepsis rate of 2%. The rate of grade 3 infections, which were largely bacterial, was 16%.

In contrast, emerging data suggests that infectious toxicity may be more prominent when InO is used in combination regimens. The COG AALL1732 study unexpectedly found during an initial safety phase that while the InO therapy blocks were well-tolerated with little reported toxicity, there was an increased risk of sepsis in subsequent myelosuppressive chemotherapy blocks, particularly DI, leading to a study amendment reducing the InO dose by 20% and recommending prophylactic administration of antibiotics during periods of neutropenia (54). A second safety phase following these changes found that the overall rate of grade 3 infections remained significantly higher on the InO arm, although rates of grade 4 and 5 infections were low and similar on both arms. The most severe infections occurred during DI Part 2, and duration of neutropenia was observed to be longer during this block on the InO Arm [27.8 days, standard deviation (sd) 12.4 days] compared to the standard arm (19.3 days, sd 8.1 days; p=0.015) (55). Based on these findings as well as increased rates of SOS during DI noted earlier, the upcoming study amendment will omit DI Part 2 on the InO arm to mitigate these risks.

Interestingly, the Alliance 041504 trial (NCT03150693) incorporating two blocks of single agent InO into a similar chemotherapy backbone for adults with newly diagnosed ALL was also suspended due to excessive toxicity; details regarding specific toxicities have not been published to date. The MDACC regimen combining InO with mini-hyper CVD for treatment of relapsed ALL included pegfilgrastim support and found that 73% of patients had grade 3 or 4 infections compared to 17% of patients treated with single agent InO in a comparison cohort (61). Data from numerous ongoing combination trials is still being collected.

### 3.4 B-cell aplasia

As CD22 is specific to B-lymphocytes, an unsurprising toxicity of InO administration is depletion of peripheral B-cells. A consistent pattern of rapid depletion followed by slow recovery of peripheral B-cells has been seen, regardless of InO dose (60).The exact time to B-cell recovery is unpredictable amongst individuals, with data on median time to B-cell recovery lacking in reports from prospective trials. Due to increased infectious toxicity among patients on the InO arm on the AALL1732 study, administration of intravenous immunoglobulin for IgG levels < 400mg/dL is recommended (54). There are functional considerations surrounding B-cell aplasia and subsequent CAR-T cell therapy that will be discussed later in this review.

## 4 Clinical trial efficacy data

Details of currently enrolling clinical trials using InO are summarized in **Table 2**.

**Table 2:** Summary of ongoing clinical trials involving inotuzumab ozogamicin*.

Trial	Sponsor	Age	Eligibility/Design	InO dose	Trial Identifier	Published results
Newly Diagnosed Patients						
InO and post-induction chemotherapy in treating patients with high-risk BCP-ALL, MPAL, and B-LLy	Children’s Oncology Group; multicenter	1 to 25 years	Newly diagnosed patients receive randomized to receive InO in consolidation phases	Two blocks of InO monotherapy given at 1.2 mg/m2 fractionated with mBFM chemotherapy backbone	NCT03959085	No efficacy data. Safety data from safety phase showed SOS in 4/25 recipients and increased rate of sepsis in delayed intensification (55)
Study of chemotherapy-free induction regimen for Ph+ ALL with InO	University of Chicago	18 years and older	Newly diagnosed and untreated patients to receive dasatinib, InO, and dexamethasone induction	Induction: Ino 1.8 mg/m2 fractionated Consolidation: If in CR/Cri, 1.5 mg/m2 fractionated. If not in CR/Cri, 1.8 mg/m2 fractionated	NCT04747912	None available
InO and combination chemotherapy in treating patients with ALL	M.D. Anderson Cancer Center	18 years and older	Unfit patients with previously untreated disease; InO combined with mini-hyperCVD and blinatumomab	0.9 mg/m2 fractionated dosing in cycle 1, 0.6 mg/m2 fractionated in cycles 2-4	NCT01371630	2-yr PFS of 58.2% and 5-yrs PFS of 44.0%. OS seen in 8% of patients (62)
Blinatumomab, InO, and combination chemotherapy as frontline therapy in treating patients with BCP-ALL	M.D. Anderson Cancer Center	14 years and older	Newly diagnosed fit patients treated with InO, blinatumomab, and hyperCVAD	0.6 mg/m2 fractionated dosing added to 2 cycles of MTX/Ara-C and to two 2 cycles of blinatumomab consolidation	NCT02877303	15 months OS in InO cohort of 87%; 10% of patients have relapsed with median follow-up of 15 months (63)
ALLTogether1- A treatment study protocol for the ALLtogether consortium for children and young adults with newly diagnosed ALL	Karolinska University Hospital, multicenter through Nordic Society for Pediatric Hematology and Oncology	0 days to 45 years	Intermediate risk patients randomized to potentially receive six doses of InO at 0.5 mg/m^2^/dose prior to start of maintenance therapy	0.5 mg/m2 dose given weekly for six weeks prior to start of standard maintenance therapy	NCT04307576	None available
Phase II study of InO induction with chemotherapy consolidation and maintenance	Goethe University, multicenter	56 years and older	Newly diagnosed unfit patients	Induction 1: 1.8 mg/m2 fractionated InO with dexamethasone and III Induction 2 and 3: 1.5 mg/m2 fractionated dosing with ITT	NCT03460522	31/42 patients (74%) patients MRD negative by end of induction 3. 2-yr OS of 91%. 1/43 patients with SOS, seen after induction 2 (64)
Relapsed/Refractory Patients						
InO for children with MRD+ CD22+ lymphoblastic leukemia	St. Jude Children’s Research Hospital	Less than 22 years of age	R/R disease with 0.1-4.99% disease	1.5 mg/m2 fractionated dosing for up to six cycles	NCT03913559	None available
Venetoclax plus inotuzumab for BCP-ALL	Dana-Farber Cancer Institute	18 years and older	Relapsed or refractory to at least 1 cycle of cytotoxic therapy	Dosing regimen not publicly available	NCT05016947	None available
InO in treating younger patients with B-LLy or relapsed or refractory CD22+ BCP-ALL	Children’s Oncology Group; multicenter	1-21 years	Primary refractory disease refractory to 2 prior induction attempts; first relapse refractory to 1 prior re-induction attempt; 2^nd^ or greater relapse. Monotherapy cohort and a cohort combining InO with mBFM chemotherapy	1.1 mg/m2 fractionated InO with standard mBFM consolidation containing cyclophosphamide, cytarabine, asparaginase, and vincristine	NCT02981628	None available for InO combined with chemotherapy
Phase I study of InO with augmented BFM re-induction for patients with R/R BCP-ALL (ALL-001)	University of Virginia; multicenter	16 to 60 years	Refractory to induction therapy or first relapse	InO dose may vary between 0.4 mg/m2 to 0.9 mg/2. Given in combination with standard doses of prednisone, vincristine, and daunorubicin	NCT03962465	None available
Phase II study of InO in patients with MRD before HSCT (ALL-2418)	GIMEMA; multicenter	18 years and older	At least two prior courses for Ph- disease; at least three months of therapy for Ph+ disease	1.5 mg/m2 fractionated InO for up to two cycles.	NCT03610438	10/39 initial patients were able to proceed to HSCT. MRD available for 20 patients, with MRD negativity seen in 7/20 (35%). Short median follow up time of 2.8 months limits outcome analysis. 1/39 (2.5%) patient with SOS, occurred prior to HSCT (65)
Phase IV single arm study of InO in R/R CD22+ BCP-ALL in China	Pfizer; multicenter	18 years and older	Failed at least one prior therapy	1.8 mg/m2 fractionated InO per cycle for up to six cycles	NCT05687032	None available
Patients following HSCT						
InO post-transplant for ALL and NHL	Case Comprehensive Cancer Center; multicenter study	16 years to 75 years	CD22+ disease between transplant days +40 and +100	Total range of dose levels is 0.1 to 0.6 mg.m2 per cycle for a maximum of 12 cycles.	NCT03104491	None available
InO and chemotherapy in treating patients with leukemia or lymphoma undergoing HSCT	M.D. Anderson Cancer Center	18 to 70 years	Patients to receive InO on transplant day -13 and as maintenance between days +45 to +100	Receive InO on days 1 and 2 of maintenance (between transplant days +45 and +100) with a second cycle between 28 days and 100 days after first cycle. InO dose not available	NCT03856216	None available

*Only studies active and enrolling as of May 31, 2023, are included.

ALL, acute lymphoblastic leukemia; B-LLy, B-cell acute lymphoblastic lymphoma; GIMEMA, Gruppo Italiano Malattie EMatologiche HSCT, hematopoietic stem cell transplant; InO, inotuzumab ozogamicin; mBFM, modified Berlin-Frankfurt; MPAL, mixed phenotype acute leukemia; MRD, minimal residual disease; NHL, non-Hodgkin lymphoma; R/R, relapsed and refractory.

### 4.1 Relapsed/Refractory

#### 4.1.1 Single agent adult studies

While the first phase I study of InO was performed in patients with R/R B-NHL, the first study in R/R BCP-ALL was a single center study at MDACC. This heavily pretreated cohort of 49 patients had a median age of 36 years and included three patients aged 16 or under. Adult patients received unfractionated InO 1.8 mg/m^2^ every 3-4 weeks while the pediatric patients received 1.3 mg/m^2^ unfractionated dosing. Fourteen percent of patients had previously undergone allogeneic HSCT. Twenty-eight patients achieved a morphologic CR; MRD was available for 27 of these with 17 (63%) being MRD negative. Among the nine patients with a CR the 12-month event-free survival (EFS) was 78% (66). The next 41 patients on this study were treated with fractionated weekly dosing in the first cycle; there was no difference in response rate based on the administration schedule but hepatic and infusional toxicity was lessened which led to this becoming the standard dosing schedule (24). A subsequent multi-center phase I/II study treated 72 patients with a median age of 45 years with 32% having previously undergone HSCT. Similar to MDACC studies, fractionated dosing of 1.8 mg/m^2^ per cycle was found to be the recommended phase II dose (RP2D). Sixty-eight percent of patients achieved CR/CRi with a median duration of response of 4.6 months. MRD was negative for 41 of 49 (84%) patients with CR/CRi. The 12-month progression-free survival (PFS) and overall survival (OS) for all patients was 20% and 30% respectively. Twenty-four patients (92% of which were in CR/CRi) proceeded to HSCT (60) Interestingly, at lower dose levels (1.2 and 1.6mg/m^2^/cycle), the CR/Cri rate was 73% and MRD negativity rate amongst patients in CR/CRi was 91%, suggesting that the RP2D may be higher than what is necessary for efficacy for some patients.

These early phase studies were followed by the pivotal phase III, randomized INO-VATE study which led to the Food and Drug Administration (FDA) approval of InO in adults with first or greater relapse of BCP-ALL. Patients were randomized to either receive InO or standard of care chemotherapy; investigators chose between fludarabine, cytarabine, and GCSF (FLAG), cytarabine plus mitoxantrone, or high dose cytarabine. InO was administered at 1.8 mg/m^2^ fractionated dosing per cycle until CR was achieved after which subsequent cycles were given at 1.5 mg/m^2^ per cycle. The CR/CRi rate for the InO arm was significantly higher than the standard chemotherapy arm at 80.7% (95% CI 72.1 to 87.7) versus 29.4% (95% CI 21.0 to 38.8). Amongst responders, the MRD negativity rate was 78.4% for the InO recipients vs 28.1% in the standard chemotherapy arm. The between-group difference was significant for all tested baseline characteristics such as disease burden, degree of CD22 expression on peripheral blasts (above or below 90%), and cytogenetics with the exception of having translocation of 4;11. There was statistically significant improvement in duration of response (DOR, 4.6 months to 3.1 months), median PFS (5.0 months to 1.8 months) and median OS (7.7 months to 6.7 months) (48). These improvements were present whether InO was given as a first or second salvage therapy (67).

For patients with Ph+ disease, CR/CRi rates, MRD negativity, and 12-month PFS were all improved for patients treated with InO although there was no OS benefit (68). The final INO-VATE follow up with a minimum of 2 years follow-up for each patient showed a 2-year OS of 22.8% for the InO group and 10.0% for the standard arm. Significantly more patients (48.2% versus 22.2%) were able to proceed to HSCT in the InO group. Amongst the InO recipients the 2-year OS for those who went to HSCT was significantly improved as compared to those without HSCT (39.4% to 13.1%). Amongst the InO recipients, variables predictive of overall survival included MRD negative remission, baseline hemoglobin > 10 g/dL, longer duration of first remission, achieving CR/CRi, and proceeding to HSCT (69). Amongst patients on the InO arm with a best response of CR/CRi, the median OS was 14.1 months for MRD negative patients vs. 7.2 months for MRD positive patients (70). Importantly, results of a multi-center, off-study, ‘real world’ retrospective analysis had outcomes similar to the INO-VATE study with a CR/CRi rate of 63% in a heavily pre-treated population (71). InO does appear to have activity against extramedullary disease outside of the CNS; in a study of 31 patients with extramedullary disease, the CR rate was 55% with a median OS of 12.8 months (42).

A phase 4 post-marketing study is evaluating a lower dose of InO (1.2mg/m^2^/cycle) in adults with R/R ALL who are eligible for HSCT and considered to have a higher risk of post-HSCT SOS (previous HSCT, salvage ≥2, age ≥55, and prior or ongoing hepatic disease). In the run-in phase of this trial, 11 of 22 patients achieved CR/CRi, of which eight had negative MRD, exceeding the threshold set to expand the trial to a larger cohort of patients randomized to either the FDA-approved dose (1.8mg/m2/cycle) compared to 1.2mg/m2/cycle. Seven patients proceeded to HSCT and two developed SOS (one grade 2, one grade 5) (72).

#### 4.1.2 Single agent pediatric studies

The first report of InO therapy in pediatrics was a retrospective analysis of five children treated at MDACC. Three of the patients received 1.3 mg/m^2^/cycle in the first cycle while two received 1.8 mg/m^2^; three patients achieved CR/CRi with one who received 1.8m/m^2^ MRD-negative (73). This was followed by a multicenter retrospective analysis of 51 pediatric patients with multiply R/R BCP-ALL treated on a compassionate access program. Any patients who received at least one dose of InO and were under 21 years of age were included. All patients were heavily pretreated with a median of five prior lines of therapy with 43% having undergone prior HSCT, 78% receiving prior CD19 directed therapy (CAR-T and/or blinatumomab) and 20% receiving prior CD22-directed therapy (CAR-T and/or moxetumomab). CR/CRi rate in this cohort was 67% with an MRD negativity rate in the responding patients of 71%. In this cohort, response was not correlated with baseline patient or disease characteristics or with number of prior relapse or prior immunotherapies/HSCT (44). The 12-month EFS and OS rates were 23.4% and 36.3% respectively. Twenty-one patients proceeded to HSCT.

Two prospective pediatric clinical trials have been performed. The ITCC-059 phase 1 study enrolled 25 patients and found the pediatric RP2D to be the same as adults (1.8 mg/m^2^/cycle in cycle 1). CR/CRi was achieved after one cycle in 60% of patients of whom 84% were MRD negative, including all ten patients treated at the R2PD. Seven patients proceeded to HSCT; three required further bridging therapy prior to HSCT. 12-month EFS and OS for the whole cohort was 28% and 40% respectively. An additional 28 patients were treated on the phase II arm of this study where the ORR was 81.5% with 18 of 22 responders (81.8%) becoming MRD negative. Eighteen patients proceeded to consolidative therapy; 14 to HSCT, two to CAR-T therapy, and two to CAR-T followed by HSCT. The 12-month EFS and OS for the 40 patients treated at the R2PD between the phase I and phase II portions of the study were 41.3% and 56.3% respectively (41). The phase II COG AALL1621 study treated 48 relapsed or refractory patients with a median of two cycles of InO at an initial dose of 1.8 mg/m^2^/cycle, with de-escalation to 1.5 mg/m^2^/cycle upon attainment of CR/CRi. After cycle 1, the CR/CRi rate was 58.3%. MRD was evaluated in 27 of the 28 responding patients and 18 were MRD negative while an additional three patients had MRD between 0.01% and 0.099%. Twenty one of 48 patients (43.8%) achieved MRD negativity by the end of cycle 2. Seventeen patients proceeded to HSCT without further bridging therapy while four proceeded after intervening chemotherapy or CAR-T therapy. In total, 14 patients received CAR-T therapy following InO. The estimated 2-year EFS and OS rates for the whole cohort were 28.6 and 36.0% respectively while being 58.8% and 68.6% respectively for patients who proceeded to HSCT or CAR-T therapy without other bridging therapy. Amongst MRD negative patients, the 2-year EFS was 57.7% (45).

Infants are a particular population of interest due to their poor outcomes as compared to older children. An international retrospective analysis reported 12 infants and three young children diagnosed at a median age of 4.4 months with 80% having *KMT2A*-rearrangement (*KMT2A*-R). Eight patients received 1.8 mg/m^2^/cycle while the rest received empirically lower dosing between 1.2 and 1.6 mg/m^2^/cycle. The CR rate was 8 of 15 (53%) with MRD negativity in 7 of 8 patients (84%). Seven patients proceeded to HSCT, three of whom were alive at last follow up. Two deaths were due to toxicity (SOS and toxic leukoencephalopathy) and the remaining 10 deaths were due to progressive disease (74).

#### 4.1.3 Combination therapy in adults

Based on promising outcomes in single agent studies, MDACC developed a clinical trial for fit adults in first salvage combining reduced intensity conventional chemotherapy (mini-hyper-CVD) concurrently with InO given in each cycle; four cycles of consolidative blinatumomab were added from patient 39 onward. To lower the risk for SOS, InO doses were decreased. Forty-eight patients were treated with a median age of 39 years. The CR/CRi/CRp rate was 92%. For responding patients, the MRD rate at any time within three cycles was 93%. The estimated 2-year progression free survival (PFS) and OS rates for the whole cohort were 42% and 54% respectively while the 1-year OS for MRD negative patients was 74% compared to 33% for MRD positive. Fifty percent of patients went to HSCT and 13 of 24 (54%) remained alive in remission after HSCT. In a propensity score matching analysis with historical controls, the overall response rate (ORR), median PFS, and median OS were significantly improved for the InO containing regimen as compared to standard chemotherapy (46). A multicenter phase I study through SWOG, S1312, combined cyclophosphamide, vincristine, and prednisone (CVP) in combination with InO in a dose escalation study. Forty-eight patients were treated; 38% had prior blinatumomab exposure, 19% patients had prior HSCT, and 10% had Ph-like disease. The MTD of InO in this combination was found to be 1.8 mg/m^2^ in cycle 1. Twenty-three patients were treated at that dose with a CR/CRi rate of 61%. Thirteen patients within the entire cohort went to HSCT and the median OS for patients treated at the MTD was 10.9 months (75).

For the special population of Ph+ ALL, MDACC performed a phase 1/2 study combining InO with the tyrosine kinase inhibitor bosutinib for patients with either R/R Ph+ ALL or lymphoid blast phase of chronic myeloid leukemia. Patients with T315I mutation were excluded. Patients were treated with 1.8 mg/m^2^ of InO in cycle 1 along with escalating doses of bosutinib; responding patients then received InO at 1.0 mg/m^2^ once every four weeks for five additional cycles. Eighteen patients (16 with ALL) were enrolled with 400 mg of bosutinib being the MTD. No SOS was seen. The CR/CRi rate was 83% with a complete molecular response occurring in 10 of 18 (56%) patients and MRD negativity in 11 of 18 (61%). Interestingly, 12 of 15 (80%) responding patients did relapse including five of six (83%) who underwent HSCT (76).

#### 4.1.4 Combination therapy in pediatrics

To date, the only data in R/R pediatric BCP-ALL comes as a phase 1b arm of the ITCC-059 study. The combination arm combined InO with a modified UKALL-R3 reinduction with vincristine and two five-day blocks of high-dose dexamethasone dosed at 20 mg/m^2^/day. At the initial InO dose level of 1.1 mg/m^2^/cycle there were liver related DLTs in two of four patients leading to a de-escalation of InO to 0.8 mg/m^2^/cycle while also amending the protocol to reduce the dexamethasone dose to 10 mg/m^2^/day. With this change the InO dose was able to be escalated serially back to 1.8 mg/m^2^/cycle which was where it was capped. The protocol initially included a plan to add asparaginase but given the observed hepatic toxicity this was not pursued. The ORR was 76.7% with 65.2% of responders achieving MRD negativity. Nine of 11 patients treated at the RP2D achieved MRD negativity. The 12-month estimated EFS was 58.1%; 14 patients proceeded directly to HSCT and six directly to CAR-T. These outcomes were similar to single agent InO but comparison is limited by small numbers (77). The COG AALL1621 study has also been amended to include a combination chemotherapy cohort where InO at 1.1 mg/m^2^/cycle is combined with an augmented BFM consolidation chemotherapy block incorporating cyclophosphamide, cytarabine, vincristine, and asparaginase; data on this approach has yet to be published. A randomized, prospective, multi-center study evaluating InO monotherapy as reinduction for pediatric patients with high-risk first bone marrow relapse compared to ALLR3 chemotherapy reinduction has been designed but is not yet recruiting (NCT04307576).

### 4.2 Clinical trials in newly diagnosed patients

#### 4.2.1 Adult trials

Based on the impressive outcomes in the R/R setting and favorable toxicity profile of single agent InO compared to intensive chemotherapy, an increasing number of studies incorporating InO into upfront therapy have been pursued, initially in older adults. A single site study at MDACC investigated the combination of InO with mini-hyper-CVD as a lower intensity induction option for adults > 60 years old with newly diagnosed BCP-ALL. InO was given on day 3 of the first four cycles at a dose between 1.3-1.8 mg/m^2^ followed by 1.0-1.3 mg/m^2^ in subsequent cycles. The final dosing schedule developed after safety analysis was 1.3 mg/m^2^ in cycle 1 and 1 mg/m^2^ for all subsequent cycles. Amongst 52 enrolled patients, the estimated 2-year PFS and OS were 59% and 66% respectively with a median PFS of 35 months. The ORR was 85%; 78% of patients were MRD negative at the time that CR was attained while 96% became MRD negative at some point within the first three cycles. Toxicity was largely hematologic and hepatic including SOS in four patients (one after HSCT) (78). Updated results of this study were recently presented providing a 5-year CR and OS rate of 76% and 46% respectively (79).

Subsequently, three separate cooperative group trials incorporating InO in combination therapy for newly diagnosed older adults have been undertaken. The German GMALL-Initial1 study combined InO at 1.8 mg/m^2^ in cycle 1 of induction with dexamethasone followed by 1.5 mg/m^2^ in cycles 2 and 3. Patients in CR proceeded with conventional consolidation, reinduction, and maintenance therapy. Forty-three patients were treated with a 100% CR/CRi rate; 53% and 74% of patients were MRD negative after the 2^nd^ and 3^rd^ induction cycles respectively. The 1-year estimated EFS and OS were 88% and 91% respectively. SOS was seen in one patient (64). The French EWALL-INO study has a two-part induction regimen following a five-day steroid prephase. In induction 1, InO at 1.8 mg/m^2^ was given in conjunction with weekly vincristine and four two-day dexamethasone pulses. Induction 2 was provided to patients with CR/CRp and incorporated InO at 1.0 mg/m^2^ with a week of dexamethasone and low dose cyclophosphamide. For patients in CR/CRp, this was followed by consolidation and maintenance with conventional chemotherapy. One hundred and thirty-one patients were enrolled with a CR/CRp rate after induction 1 of 88.5% with MRD negativity in 56.6% of tested responding patients which increased to 80.8% after induction 2. The 1-year leukemia-free survival and OS were 64.9% and 72.5% respectively. Three patients developed SOS (80). The Alliance A041703 study (NCT03739814) is investigating a completely chemotherapy-free induction regimen for elderly patients, giving InO for induction (initial dosing 1.8 mg/m^2^ fractionated) followed by blinatumomab consolidation. Amongst the first 33 treated patients, the CR rates after initial InO induction and follow up blinatumomab course were 85% and 97% respectively. The 1-year EFS and OS were 75% and 84% with a median follow-up of 22 months. There were 12 events including nine relapses, two deaths in remission, and one death without remission due to SOS (81).

Studies are ongoing incorporating InO with more intensive chemotherapy regimens for younger fit patients. A phase II study at MDACC for patients aged 18-59 years with conventional hyper-CVAD followed by 4 cycles of blinatumomab was subsequently modified to incorporate InO: Sixty-two patients were treated and beginning with patient 39, InO at a dose of 0.6 mg/m^2^/cycle (0.3mg/m^2^/dose on days 1 and 8) was added to two of the methotrexate/cytarabine hyper-CVAD cycles and two of the blinatumomab cycles. All patients achieved a CR and 91% achieved MRD negativity. For the whole cohort the 3-year continuous remission duration and OS were 83% and 84% respectively. Amongst InO recipients, there was only one relapse and no deaths. No SOS was reported (82). The median duration of follow up on this study was short at 23 months. The multicenter Alliance A041501 study, a phase III study for newly diagnosed patients aged 18-39 years, investigated the addition of two cycles of single agent InO at 1.5mg/m^2^/cycle into an intensive pediatric-inspired chemotherapy backbone; the two InO cycles were given back-to-back following a 4-drug induction, and then followed by Consolidation, Capizzi-style methotrexate Interim Maintenance (IM), Delayed Intensification (DI), and Maintenance phases. As noted earlier, this trial was suspended early to unacceptable toxicity noted in the later intensive chemotherapy blocks, particularly DI.

#### 4.2.2 Pediatric trials

In pediatrics, the COG AALL1732 study is an ongoing phase III trial assessing the safety and efficacy of incorporating InO into post-induction therapy for patients aged 1-24 years with high-risk features. In the initial design, patients received a four-drug induction followed by augmented BFM Consolidation. Patients with MRD <0.01% by flow cytometry by the end of Consolidation and CD22 expression on >20% of blasts at diagnosis were eligible to be randomized to either chemotherapy alone or chemotherapy plus two cycles of InO at 1.5mg/m^2^/cycle intercalated between Consolidation and the start of a high-dose methotrexate (HD-MTX) based IM1 course and between IM1 and DI. After an initial safety phase, the dose of InO was reduced to 1.2mg/m2/cycle and additional anti-infective supportive care measures were added (54). A second safety phase following these changes revealed ongoing concerns regarding risk of infection and SOS, particularly during the DI block, as detailed earlier in this review. The study is being amended to further decrease the InO dose to 0.9mg/m^2^/cycle for the second InO course and to replace the toxic chemotherapy block (DI Part 2) with InO Block 2 with the goal of mitigating toxicity while improving efficacy (55). The AllTogether1 frontline trial (NCT 04307576) is evaluating the addition of six weekly doses of InO at 0.5mg/m2/dose after completion of intensive chemotherapy and prior to Maintenance in patients with IR-High risk stratification. These ongoing trials will be critical to determining the optimal dosing strategy that balances risk of toxicity with improved outcomes.

### 4.3 InO for MRD clearance

An additional area of exploration is using InO in low-level disease states to achieve an MRD negative remission. A single-arm phase II trial at MDACC enrolled patients who were in CR but not MRD negative after at least 3 months of frontline therapy or who had MRD-positive relapse. Patients received InO at a reduced dose of 0.9 mg/m^2^/cycle in cycle 1 and 0.6 mg/m^2^ of cycles 2-6. Twenty-seven patients were treated and 67% became MRD negative, the vast majority of whom did so after cycle 1. With a median follow up of 18 months, 14 of 18 (78%) responding patients remained MRD-negative; the RFS and OS for this group was not reached. Five of 18 (28%) responders underwent HSCT. Two patients developed SOS during InO therapy, one after 1 month and one during cycle 5 (83). The multicenter Italian GIMEMA ALL2418 study investigated using InO to obtain MRD negativity as a bridge to HSCT. Patients received InO at a dose of 1.5 mg/m^2^/cycle for one cycle; patients who did not achieve MRD negativity could receive a second cycle at the same dosing. Responding patients received either low-dose chemotherapy (alternating vincristine, cyclophosphamide, prednisone, methotrexate, and mercaptopurine) if Ph-negative or TKI if Ph+ for up to 12 weeks to allow for InO washout prior to HSCT. In a report of the first 39 patients treated, MRD was available for 20 patients of whom seven (35%) achieved MRD negativity. Ten patients (26%) underwent HSCT including the seven with negative MRD, one with low level MRD, and two with not yet available MRD. Median OS was not reached but median follow up was very short at 2.8 months. One case of SOS was seen 20 days from last dose of InO and before HSCT (65). A pediatric study evaluating InO for treatment of MRD-positive disease is enrolling at St. Jude Children’s Research Hospital, but data has not been presented (NCT03913559). These studies suggest InO may be an effective and potentially safe bridge to HSCT although SOS was infrequently seen even at lower InO doses; further studies systematically evaluating lower doses are needed to fully quantify the balance between efficacy and toxicity.

### 4.4 InO in the peri-HSCT setting

InO is being evaluated in the peri-HSCT period in adults as a means to reduce relapse in two clinical trials. A multicenter phase I patients enrolled patients aged 16-75 who were in CR after HSCT and at high risk for relapse, defined as: MRD-positive before or after HSCT, in second CR or beyond, recipient of reduced intensity conditioning, lymphoid blast crisis of CML, or Ph-like ALL. Patients needed to have adequate graft function, no grade III/IV GVHD, no active any grade liver GVHD, and no history of SOS. InO was to be given in 28-day cycles as a single dose per cycle for up to 12 cycles starting between day +40 and day +100 after HSCT. Eighteen patients were treated with a median follow-up of 18.1 months. The median time to first dose was 84 days after HSCT. The R2PD was 0.6 mg/m^2^ with one DLT of prolonged thrombocytopenia. PFS and OS at 1-year post HSCT was 88.9% and 94.4% with one death (due to GVHD) and one relapse at 7-month post-HSCT. No SOS was observed (84). A similar study, also incorporating a dose of InO on transplant day -13, is open at MDACC. While intriguing, larger and randomized studies will be necessary to determine if this approach is truly beneficial in terms of reducing relapse risk.

## 5 Predictors of response and resistance

### 5.1 Cytogenetic features

Univariate analysis of a retrospective cohort of 89 adult patients identified complex karyotype, translocation (4, 11), translocation (9, 22), and abnormal chromosome 17 as risk factors for failing to achieve a CR; this group had median OS of 5.0 months compared to 44 months for all other patients (85). On INO-VATE, 284 of 326 (87%) randomized patients had cytogenetic data available. CR/CRi rates and MRD negativity rates for patients treated with InO were similar between patients with diploid, Ph+, complex, and other cytogenetic types. However, the DOR was different between subgroups, with a median of 7.0 months for the diploid group, 4.2 months for the complex, 5.9 months for Ph+, and 8.0 months for others. It was notably shorter for patients with *KMT2A*-R, but the sample size was small with only eight patients. PFS followed a very similar pattern by subgroup although there were no significant differences in OS between the groups (86). In pediatrics, responses have been reported in high-risk genetic subgroups such as hypodiploidy, *KMT2A-*R, and Ph-like ALL (44). In the largest mature prospective pediatric study, AALL1621, there was no association between cytogenetics and outcomes, but analysis was limited by sample size (45). Of note, on AALL1621 and in the retrospective analysis of infants treated with InO, zero of six (0%) and two of 12 (17%) patients with *KMT2A*-R respectively achieved MRD negativity, suggesting this may be a group with some inherent insensitivity to InO (45, 74).

### 5.2 CD22 expression

The leukemic blast population in an individual patient may be heterogeneous in terms of sCD22 expression which may impact response to CD22-targeting therapies like InO as sCD22 expression on leukemic blasts is required for InO activity (11). Positive expression of sCD22 is defined as > 20% above background, with background being defined as 1% positivity on an appropriate negative control population; the number of sCD22 molecules may also vary, resulting in reports of expression ranging from dim to bright. Flow cytometric evaluation of sCD22 can be technically challenging, requiring an experienced laboratory and use of a bright fluorophore (e.g., phycoerythrin) to optimize detection (87).

The impact of variations in sCD22 expression on InO response has shown mixed data in clinical trials. The INO-VATE trial found that response rates with InO were superior compared to conventional chemotherapy regardless of sCD22 expression using a cutoff of 90% of blasts expressing sCD22 (48). For patients with >90% sCD22 positivity, the CR/CRi rate was 78.5% in InO recipients vs 35.5% on the standard chemotherapy arm; for patients with <90% sCD22 positivity, the CR/CRi rates were 65.7% and 30.6% respectively. MRD negativity rates, DOR, EFS, and OS followed a similar pattern. Surface CD22 receptor density was evaluated by assessing molecules of equivalent soluble fluorochrome (MESF). When dividing patients into quartiles by MESF measurement, the quartile with the lowest sCD22 receptor density appeared to have the least benefit from InO as compared to chemotherapy suggesting response to InO may be somewhat limited in patients with lower sCD22 receptor density (88), a result seen in additional subsequent adult cohorts (89).

Notably, when patients relapsed after an initial response to InO on INO-VATE, a decrease in sCD22 positivity and MESF compared to baseline was seen in 90% of patients. For InO recipients who were MRD-positive at the end of therapy, sCD22 decreased from being present on a median of 98.4% of blasts to 45.2% (88). Similar data was seen for pediatric patients on AALL1621, where eight of 13 (62%) non-responders had a decrease in sCD22 expression from baseline and all four patients with baseline sCD22 <90% had residual disease after cycle 1 with predominantly sCD22-negative populations. CD22 receptor density as assessed by CD22 antibody bound per cell did not change significantly (45). While baseline CD22 density was lower for non-responders (1022 sites per cell, range 290-8848) compared to responders (4123 sites per cell, range 762-10715), there was no site density threshold that predicted lack of response. These data suggest that either subpopulations of sCD22 negative blasts present at the time of InO therapy escape therapy and preferentially expand, or that sCD22-positive blasts may downregulate CD22 expression in response to InO therapy as a mechanism of resistance. In addition, while there may be a trend toward lower likelihood of response with “dim” sCD22 expression, “dim” expression does not preclude response to InO. In contrast to the AALL1621 findings, on the pediatric ITCC-059 study there was no difference between InO responders and non-responders with regard to sCD22 positivity, CD22 receptor density, the level of CD22 saturation on peripheral blasts, nor the degree of InO internalization after dosing (41, 51). Of note, on AALL1621, four of six patients with *KMT2A*-rearrangment had central flow cytometric evaluation of sCD22; three had partial sCD22 expression at baseline and all had CD22 site density < 1500 sites per cell, suggesting that variable sCD22 expression, particularly negative subpopulations, may account for the reported inferior responses for this cytogenetic subgroup (45).

### 5.3 CD22 splicing

In a phase I trial of CD22-directed CAR-T cells, the downregulation of CD22 protein levels did not fully correlate with decreases in *CD22* mRNA levels which could suggest post-transcriptional means of protein degradation are important in immunotherapy settings (90). Accordingly, a single patient case report used digital droplet PCR to identify a novel *CD22* truncating mutation that directly led to decreased CD22 expression in a patient who initially responded to InO prior to relapsing with CD22-negative disease (91). In a study where RNA sequencing was performed on 219 pediatric BCP-ALL samples, five isoforms that skipped exon 2 were identified. *In vitro*, exon 2 skipping precluded *CD22* mRNA translation and led to decreased sensitivity to InO similar in degree to what was seen with complete *CD22* knockout (92).

Interestingly, in samples from patients on AALL1621, there was a correlation between CD22 cell surface expression with the expression of *CD22* exon 2 including isoforms but not total transcript levels, suggesting that the inclusion of exon 2 is critical for protein expression. Additionally, one patient who at baseline only expressed an exon 2 skipping isoform failed to respond to InO. Finally, in one patient with disease progression and downregulation of CD22 after InO treatment, there was a significant shift in isoforms toward exon 2 skipping isoforms. Taken together, these data suggest that alternative splicing is important for both response and resistance to InO. However, on the ITCC-059 study, no correlation between exon 2 skipping and clinical response was seen (41).

### 5.4 Alterations in apoptotic pathways

Pre- and post-treatment samples from 28 patients on AALL1621 were profiled by customized mass cytometry by time of flight (CyTOF) to identify prognostic biomarkers for response. While patients with CD22^high^ expression were more likely to achieve CR/CRi, response was also seen with patients with CD22^low/intermediate^ expression, highlighting that CD22 levels alone are not a comprehensive biomarker. Bcl-2 expression was found to be prognostic in combination with CD22, such that patients who did not achieve CR/CRi had high frequency of cells with CD22^low^Bcl-2^high^ phenotype whereas the inverse phenotype was seen at higher frequency in responders. Additionally, patients with either failure to achieve CR/CRi or MRD negativity had post-treatment blasts with expression of anti-apoptotic proteins such as Bcl-2, Bcl-xL, and/or Mcl-1 (93). Taken together with pre-clinical data showing synergy between InO, dexamethasone, and the Bcl-2 inhibitor venetoclax in murine patient-derived xenograft (PDX) models of BCP-ALL, these data suggest that combining InO with venetoclax may be a strategy to improve responses (94). A clinical trial using InO, venetoclax, and dexamethasone in adult patients with relapsed BCP-ALL is currently open and enrolling (NCT05016947).

## 6 Practical considerations

### 6.1 Consideration of CAR-T as an alternative CD22 targeting therapy

Multiple early phase studies have been published investigating the use of CD22 targeting CAR-T cells in patients with R/R BCP-ALL. Both CAR-T and InO require sCD22 expression and both can plausibly be used in patients with downregulation of other targetable antigens such as CD19. Potential benefits to CAR-T therapy compared to InO include the lack of published evidence of SOS and the ability of CAR-T cells to treat extramedullary disease (95, 96). Fifty-eight patients were treated on the largest pediatric study of CD22 CAR-T therapy. The CR rate was 70% for this heavily pretreated population in which 67% of patients had prior HSCT and 62% had prior CD19 CAR-T exposure. The median OS was 13.4 months and 13 patients were able to proceed to consolidative HSCT. Interestingly, hemophagocytic lymphohistiocytosis-like toxicity (carHLH) was seen in 32.8% of patients receiving that CAR construct (16). An additional study in pediatrics had clinical response in only one out of three patients although changing the CAR construct to include a short-linker CD22 single chain variable fragment improved function in pre-clinical studies (97). Dual CD19/CD22 CAR-T trials have also been published with promising results in pediatrics although engineering improvements are needed to create constructs without *in vivo* predominance for targeting CD19 over CD22 (98–100).

Direct comparisons between InO and CD22 CAR-T therapy are difficult due to differing study designs and cohorts, although efficacy appears similar. Both agents are primarily bridges to subsequent consolidative HSCT for patients with R/R disease. CD22 downregulation as a resistance mechanism can occur with either (16). However, CD22 CAR-T is currently only available in a select few centers as an investigational agent, as there are no FDA approved products. Additionally, CD22 CAR-T requires T-cell collection followed by at least a few weeks of manufacture which often requires bridging chemotherapy. In contrast, InO is available commercially and can be administered as an outpatient IV infusion on short notice without lag time. For these reasons, we consider InO to be the first line CD22 targeting therapy with CD22 CAR-T being reserved for patients in need of subsequent retrieval who maintain CD22 expression, or patients considered to be at very high risk for HSCT-related SOS (i.e., history of SOS or significant liver disease). CD22 CAR-T remains an area of active investigation so this recommendation may change over time.

### 6.2 Planned curative consolidation following InO for patients with relapsed/refractory disease

Multiple studies previously mentioned herein have demonstrated that long term survival after InO therapy is maximized by consolidative therapy. The bulk of the data is on consolidative HSCT but there is also increasing use of InO as bridge to CD19 CAR-T therapy.

The risk of SOS, particularly after consolidative HSCT, has been consistently seen across multiple studies in both adults and children. Therefore, close coordination between the oncologist and HSCT team/center regarding the timing and use of InO is important. While data has not strongly implicated time from last InO dose to HSCT as a consistent risk factor for subsequent SOS development, it has been recommended to delay as long as capable whilst still maintaining maximal remission (44, 52); for gemtuzumab ozogamicin (GO), the risk of SOS was reported to be increased in patients who proceeded to transplant within 3 months of receiving GO (101). Since InO dose exposure is associated with risk for SOS, minimizing the number of doses may be a mechanism to limit SOS incidence and frequent marrow assessment after individual doses to assess response rather than empirically administering full courses could be considered in select patients for whom HSCT at a short interval is planned (27). Use of dual alkylator conditioning regimens should be avoided due to increased SOS risk on multivariate analysis while single center data suggests an increased risk when using sirolimus for GVHD prophylaxis (52, 53). The benefit of ursodiol prophylaxis is not established but it is a safe medication with minimal toxicity so is generally recommended both during InO treatment and during HSCT (57). Largescale data on prophylactic defibrotide has not demonstrated benefit and small series provide inconsistent results, but this can be considered, especially for patients deemed to be at significantly high risk. Ongoing clinical trials will address whether lower InO dosing will result in adequate MRD-negative remission rates, particularly when combined with chemotherapy, while also decreasing rates of SOS with subsequent HSCT. Research to identify biomarkers predictive of SOS risk will be critical to improving outcomes for patients receiving HSCT after InO therapy.

Coordination between treating oncologist and CAR-T team/center is also encouraged regarding the decision to use InO as bridging therapy prior to CD19-targeting CAR-T therapy. There is data to suggest that the presence of B-cell antigen (whether on malignant B-lymphoblasts, normal B-lymphocytes, or both) is necessary for maximal CAR-T expansion (102). InO exposure typically leads to a period of B-cell aplasia, the duration of which is not predictable across patients. Thus, patients with MRD-negative disease response to InO will have little to no malignant or normal B-cell antigen available to CAR-T targeting and expansion for some interval, typically weeks, after InO exposure. There is not a clear consensus on whether CAR-T infusion should be delayed in patients with very low disease burden after InO until any B-cell recovery is demonstrated. Outcomes for CAR-T recipients who are MRD negative at the time of infusion are better than for patients with detectable disease, although many of those patients do have higher normal B-cell numbers if they were bridged using conventional chemotherapy (103). A recent multicenter retrospective analysis of 39 pediatric patients who received InO as bridging therapy prior to CD19 CAR-T therapy revealed similar day 28 response rates and 12 month EFS and OS compared to previously published reports of pediatric patients treated with CD19 CART without prior InO exposure (15, 104). However, in this analysis, delay in CAR-T infusion until evidence of B-cell recovery was variable.

### 6.3 Mitigating infectious toxicity

Infectious risk after InO is likely increased both due to myelosuppression and hypogammaglobulinemia from B-cell depletion. This is especially pronounced when InO is combined with conventional chemotherapy (as compared to InO monotherapy). For example, the infection rate in the phase II adult study combining InO with mini-hyper-CVD in fit adults was 71% as compared to 17% in monotherapy study (105). As seen in the safety phases of the upfront AALL1732 study, this can lead to increased toxicity even in subsequent phases of therapy following InO administration (54). While guidelines from the European Conference on Infections in Leukemia (ECIL-9) did not recommend routine antibiotic prophylaxis for InO recipients, the COG does recommend prophylactic antibiotics during periods of neutropenia for pediatric patients on studies with where InO is combined with multiagent chemotherapy regimens (105). Additionally in settings with access to IVIG, IgG levels should be monitored with repletion recommended for levels under 400.

### 6.4 How we approach relapse given the availability of various active agents

Promising outcomes in relapsed pediatric BCP-ALL have been seen in recent years with immune-based therapies including blinatumomab, tisagenlecleucel, and InO. However, there are no studies directly comparing these agents and no formal guidelines on which agents to use in which clinical context.

When deciding on which agent to use, we account for the number of relapses, disease burden, sites of disease, prior therapies including HSCT, current and prior toxicities, and subsequent therapy options. Obviously if patients have CD19 or CD22-negative blasts, this will drive treatment selection. In the first marrow relapse setting, use of blinatumomab is the primary strategy, as demonstrated by the results of the COG AALL1331 trial. For patients with first late marrow relapse (≥36 months from initial diagnosis) and MRD <0.1 after intensive reinduction, continuation therapy combining blinatumomab and conventional chemotherapy provided a 4-year DFS of 72.7% (106). Patients with early first marrow relapse or persistent MRD after reinduction had superior outcomes with blinatumomab consolidation as a bridge to HSCT, and is considered optimal therapy for this patient population (14), although tisagenlecleucel either as standalone therapy or as bridge to HSCT may also be considered. In contrast, on AALL1331, patients with isolated extramedullary disease had an outcome much poorer than historical trials and no benefit from blinatumomab, highlighting the lack of efficacy of blinatumomab for treatment or prophylaxis of CNS leukemia. Notably, anecdotal reports also raise concern about the efficacy of blinatumomab for non-CNS extramedullary disease (107). InO also is unable to cross the blood-brain barrier but does have some activity reported in non-CNS extramedullary disease (30, 42). In contrast, CAR-T therapy has been demonstrated to have significant activity for extramedullary sites, including the CNS, and is a preferred treatment approach for such patients (108). One challenge, however, is that tisagenlecleucel is not FDA-approved for first CNS relapse. Clinical trials of CAR-T cell therapies in first CNS relapse are urgently needed, given the potential to spare responding pediatric patients the long-term toxicities associated with cranial radiation.

For patients with second or greater relapse or refractory disease, tisagenlecleucel has the benefit of being potentially curative in some cases without subsequent HSCT, while both blinatumomab and InO are optimally used to achieve deep MRD-negative remission as a bridge to HSCT. For patients with prior HSCT, tisagenlecleucel is typically the therapy of choice for this reason. However, tisagenlecleucel does require patient stability for T cell collection and during manufacturing, and bridging therapy is typically required. Many providers may avoid the use of blinatumomab prior to tisagenlecleucel due to concern for targeting the same antigen (CD19) and risk of downregulation that could impact tisagenlecleucel activity. Data addressing this concern demonstrate that prior blinatumomab exposure does not appear to impact response to tisagenlecleucel unless patients were refractory to blinatumomab (43). InO is an appealing bridging agent for patients in whom consolidation with tisagenlecleucel is planned since it targets a different antigen and does not seem to negatively impact T-cell collection (104), although consideration must be given to timing of CAR-T therapy after InO given the potential for transient eradication of normal and malignant B cells as previously discussed. Due to SOS concerns with HSCT after InO, if the optimal treatment for a patient is to rapidly proceed to a consolidative HSCT, InO may be less ideal than blinatumomab. In contrast, for a patient with prior significant neurotoxicity, the risk of ICANs may be taken into consideration.

InO also has the benefit of high efficacy in the setting of high disease burden whereas outcomes for tisagenlecleucel and blinatumomab are better when used in low disease/MRD settings, which suggests an important role for InO in initial debulking (103, 109). This is of particular interest in patients with early first relapse, as well as multiply relapsed patients, who respond very poorly to retrieval attempts with conventional chemotherapy (14). Future trials in the early first relapse setting will evaluate InO compared to intensive chemotherapy, followed by blinatumomab to eradicate MRD and provide spacing between InO therapy and subsequent HSCT in an effort to decrease risk of SOS.

## 7 Future directions

Success with InO as well as other targeted agents such as blinatumomab, venetoclax, and for Ph+ ALL, tyrosine kinase inhibitors (TKI) raises the possibility of replacing components of conventional cytotoxic chemotherapy from BCP-ALL regimens with novel targeted agents including InO. The Alliance A042001 randomized phase II trial (NCT05303792) will investigate whether the experimental arm (InO in combination with mini-hyperCVD) results in superior MRD-negative EFS compared with a control arm of dose-adjusted hyperCVAD in previously untreated adults aged 50 years or older with CD22+ Philadelphia chromosome–negative (Ph-) BCP-ALL. The COG AALL1732 trial is being amended to omit the second half of Delayed Intensification, the block of conventional chemotherapy with the greatest toxicity including SOS, myelosuppression, and infections, and replace this with one of two blocks of InO. Truly chemotherapy-free regimens are also under investigation in fragile populations. The Alliance A041703 phase II open-label multicenter clinical trial (NCT03739814) is evaluating the efficacy InO induction followed by blinatumomab as initial treatment for newly diagnosed adult patients ≥ 60 years (81), and the COG is considering a similar approach in children with Down syndrome.

## 8 Conclusions

In conclusion, InO has demonstrated significant efficacy in the R/R setting in both adults and children as compared to conventional chemotherapy regimens. Burgeoning data in adults suggests InO may be beneficial as part of combination therapy in newly diagnosed patients, however the optimal approach that balances efficacy with toxicity risk remains to be established. A large-scale prospective randomized pediatric trial in the upfront setting is ongoing although efficacy data has not yet been presented. Toxicities such as myelosuppression, hypogammaglobulinemia, and SOS (particularly after subsequent HSCT) have been consistently seen across trials. Improved understanding of risk factors for SOS beyond subsequent HSCT including identification of predictive biomarkers and/or pharmacogenomic alleles will be critical to safely incorporating InO into both frontline and relapsed regimens. Mitigation measures to lower the risk for SOS would be useful, although any benefit of prophylactic ursodiol and defibrotide has not been established, and other potential interventions for high-risk patients have not been identified. Areas of ongoing and needed exploration include finding the optimal dosing regimen in combination regimens as well as combining InO with other targeted agents.

## Author contributions

JR and MO’B contributed to conception and design of this review. JR wrote the first draft of the manuscript and created the figure and table. MO’B wrote sections of the manuscript. All authors contributed to the article and approved the submitted version.
